# Supplementary figures and images for: An old drug and different ways to treat cutaneous leishmaniasis: Intralesional and intramuscular meglumine antimoniate in a reference center, Rio de Janeiro, Brazil
Source: PLoS Negl Trop Dis. 2021 Sep 23;15(9):e0009734. doi: 10.1371/journal.pntd.0009734 (PMC8491910; doi:10.1371/journal.pntd.0009734)

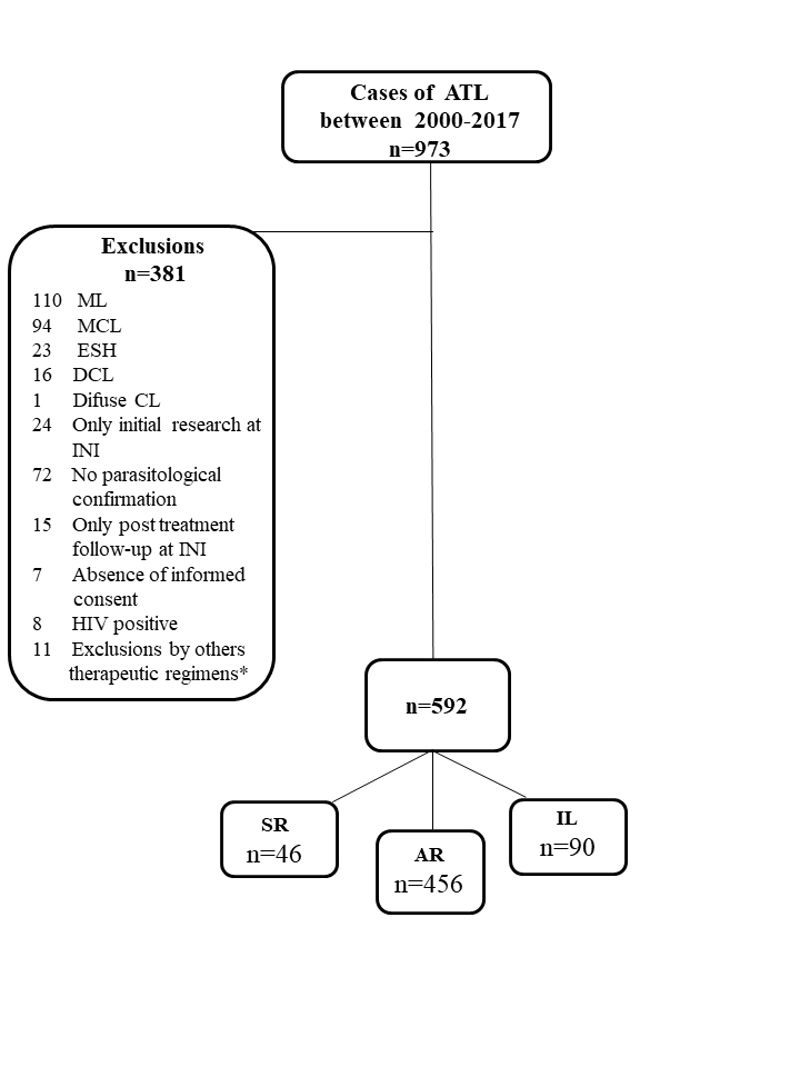

Supplement: S1 Fig — MA- meglumine antimoniate; INI- Evandro Chagas National Institute of Infectious Diseases; Fiocruz- Oswaldo Cruz Foundation; ML- mucosal leishmaniasis; MCL- mucocutaneous leishmaniasis; ESH- early spontaneous resolution without treatment; DCL- disseminated cutaneous leishmaniasis; CL- cutaneous leishmaniasis; HIV- human immunodeficiency virus; LCL- localized cutaneous leishmaniasis; SR- standard regimen with MA 10 to 20 mg / kg / day; AR- alternative regimen with MA 5 mg / kg / day; IL- intralesional MA. *MA used as first therapeutic regimen. (TIF) [file pntd.0009734.s001.tif]

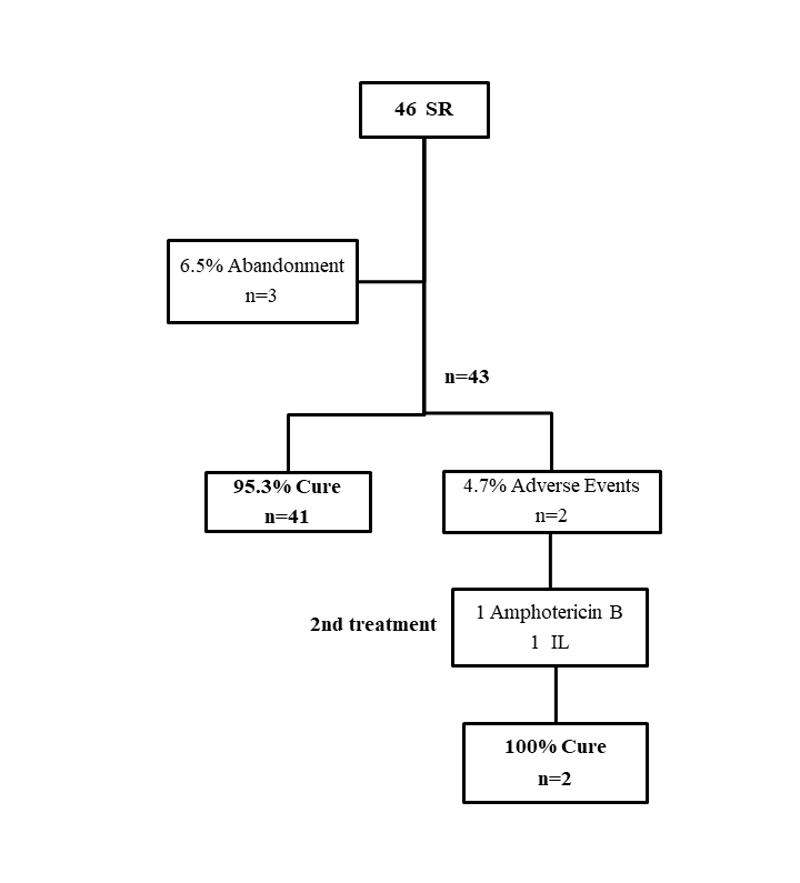

Supplement: S2 Fig — SR- standard regimen; IL- intralesional route. (TIF) [file pntd.0009734.s002.tif]

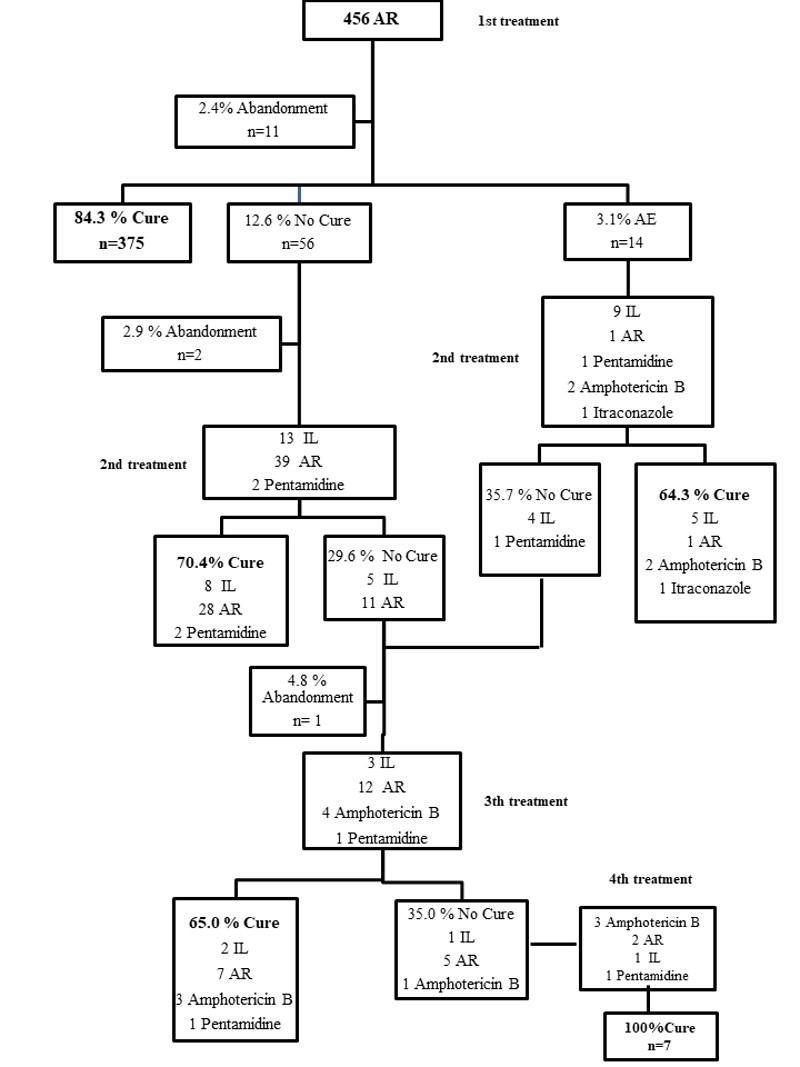

Supplement: S3 Fig — AR- alternative regimen; SR-standard regimen; IL- intralesional route; AE—adverse events. (TIF) [file pntd.0009734.s003.tif]

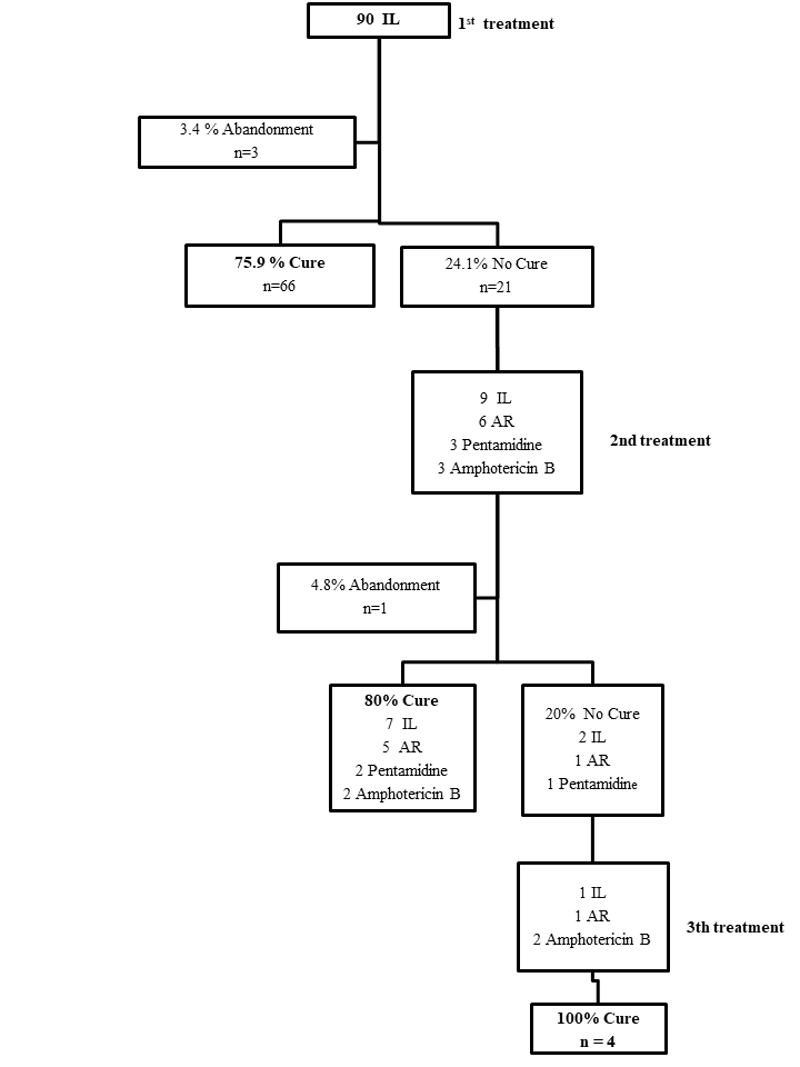

Supplement: S4 Fig — IL- intralesional route; AR- alternative regimen; SR- standard regimen. (TIF) [file pntd.0009734.s004.tif]

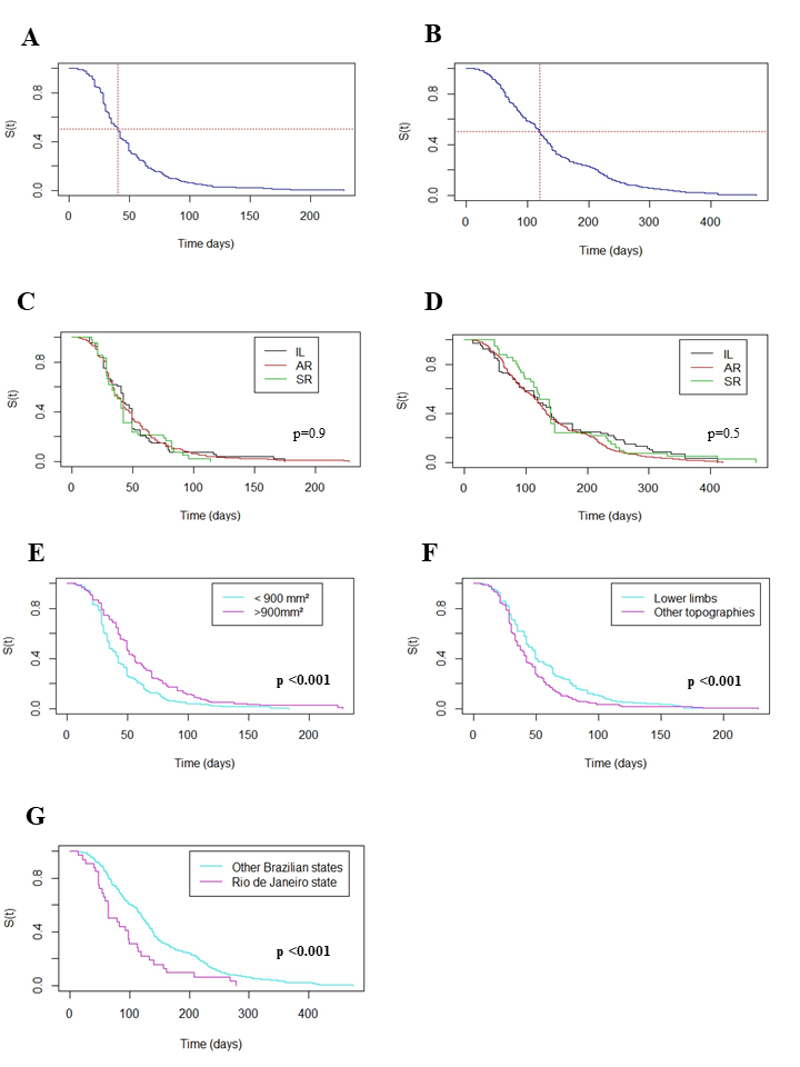

Supplement: S5 Fig — A) Epithelialization of cutaneous lesions; B) Complete healing of cutaneous lesions; C) Epithelialization of lesions according to the groups; D) Complete healing according to the groups; E) Epithelialization according to lesion´s area; F) Epithelialization according to lesion topography; G) Complete healing according to likely location of infection. SR- standard regimen; AR- alternative regimen; IL- intralesional route; S(t)- Survival function. Bold: p-value <0.05 (according to Log-Rank test). (TIF) [file pntd.0009734.s005.tif]

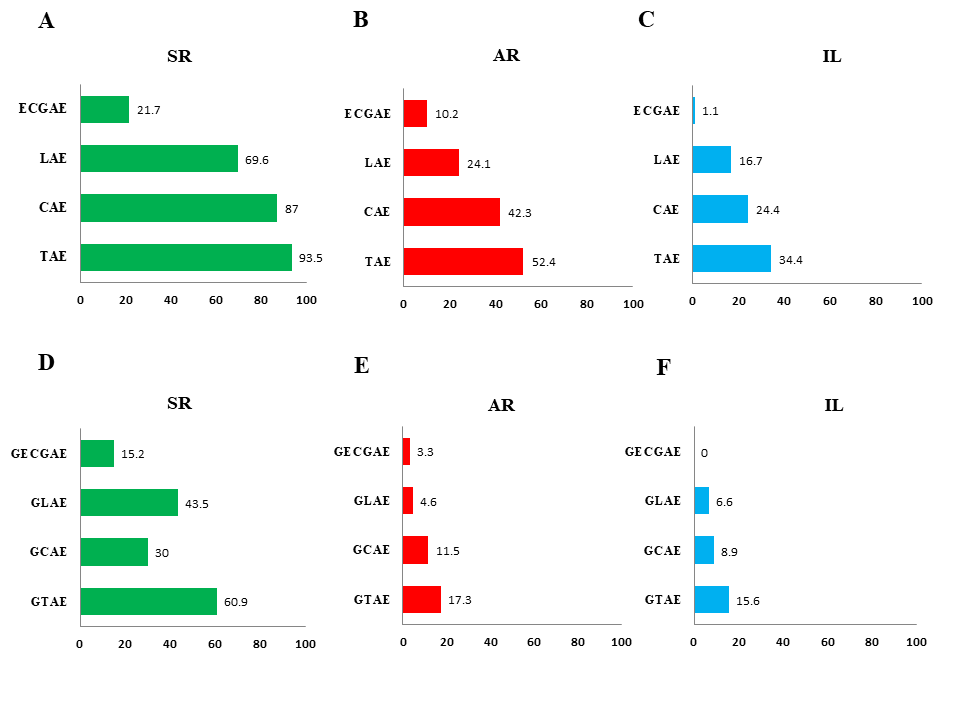

Supplement: S6 Fig — A) Number of cases of SR group with the occurrence of adverse events; B) Number of cases of AR group with the occurrence of adverse events; C) Number of cases of IL group with the occurrence of adverse events; D) Proportion of cases of SR group with moderate to severe adverse events, compared to the other groups; E) Proportion of cases of AR group with moderate to severe adverse events, compared to the other groups; F) Proportion of cases of IL group with moderate to severe adverse events, compared to the other groups. TAE- total adverse events; CAE- clinical adverse events; LAE- laboratory adverse events; ECGAE- Electrocardiographic adverse events; GTAE- grouped (moderate to severe) total adverse events; GCAE- grouped (moderate to severe) clinical adverse events; GLAE- grouped (moderate to severe) laboratory adverse events; GECGAE- grouped (moderate to severe) electrocardiographic adverse events; SR- standard regimen; AR- alternative regimen; IL- intralesional route. (TIF) [file pntd.0009734.s006.tif]
