# Supplementary material for: An old drug and different ways to treat cutaneous leishmaniasis: Intralesional and intramuscular meglumine antimoniate in a reference center, Rio de Janeiro, Brazil
Source: PLoS Negl Trop Dis. 2021 Sep 23;15(9):e0009734. doi: 10.1371/journal.pntd.0009734 (PMC8491910; doi:10.1371/journal.pntd.0009734)
Supplement: S1 File — SR- standard regimen; AR- alternative regimen; IL- intralesional route. (DOCX) [file pntd.0009734.s007.docx]

**Outcomes in subsequent treatments**

| **First Treatment** | **N=592** | **n/N*** | **Cure rate (%)** |
| --- | --- | --- | --- |
| **SR** | 46 | 41/43 | 95.3 |
| **AR** | 456 | 375/445 | 84.3 |
| **IL** | 90 | 66/87 | 75.9 |

| **Second treatment** | **N=91** | **n/N*** | **Cure rate (%)** |
| --- | --- | --- | --- |
| **AR** | 46 | 34/46 | 73.9 |
| **IL** | 32 | 21/32 | 65.6 |
| **Amphotericin B** | 6 | 5/5 | 100 |
| **Pentamidine** | 6 | 4/6 | 66.7 |
| **Itraconazole** | 1 | 1/1 | 100 |

| **Third treatment** | **N=24** | **n/N*** | **Cure rate (%)** |
| --- | --- | --- | --- |
| **AR** | 13 | 8/13 | 61.6 |
| **IL** | 4 | 3/4 | 75 |
| **Amphotericin B** | 6 | 5/6 | 83.3 |
| **Pentamidine** | 1 | 1/1 | 100 |

| **Fourth treatment** | **N=7** | **n/N*** | **Cure rate (%)** |
| --- | --- | --- | --- |
| **AR** | 2 | 2/2 | 100 |
| **IL** | 1 | 1/1 | 100 |
| **Amphotericin B** | 3 | 3/3 | 100 |
| **Pentamidine** | 1 | 1/1 | 100 |

SR- standard regimen; AR- alternative regimen; IL- intralesional route.

***considering abandonment rate**
