# Supplementary material for: An old drug and different ways to treat cutaneous leishmaniasis: Intralesional and intramuscular meglumine antimoniate in a reference center, Rio de Janeiro, Brazil
Source: PLoS Negl Trop Dis. 2021 Sep 23;15(9):e0009734. doi: 10.1371/journal.pntd.0009734 (PMC8491910; doi:10.1371/journal.pntd.0009734)
Supplement: Attachment — Submitted filename: Response_to_Reviewers.docx [file pntd.0009734.s008.docx]

**COMMENTS, RESPONSES AND CHANGES**

**REVIEW COMMENTS:** An old drug and different ways to treat cutaneous leishmaniasis: intralesional and intramuscular meglumine antimoniate in a reference center, Rio de Janeiro, Brazil

**GUIDANCE FOR THE REVIEWER:** Each comment has an answer and the page number of the manuscript where it is located.

**GENERAL COMMENTS:**

**1.** Statistical analysis was better explained in the abstract and in the methods.

**CHANGES TO FIGURES AND TABLES**

**1.** Corrections on Figure 1.

**2.** We changed the term “failure” to “no cure” in Figures 3 and 4.

**3.** Corrections on Figure 6.

**4.** Corrections on Table 1.

**5.** Additional data supporting files (S1 File and S1 Data).

----------------------------------------------------------------------------------------------------------

**REVIEWER 1**

**1. METHODS**

**Comment 1.1:** The objectives of this study are clearly defined, however there is no power calculation discussed to determine if the groups in each arm meets statistical significance.

**Response 1.1:** We would like to explain the reason why we did not calculate the sample sizes before the begining of the study, mainly because of the observational design and the clinical practice. Although we did not conduct the study as a clinical trial, we highlight that this cohort followed an expressive number of patients from a reference centre for infectious diseases during the clinical practice. We thank for the suggestion. We calculated the study power based on the estimations obtained in this sample. Assuming that the main objective was to compare the cure proportion among the different treatments, we used the 5% significance level, the proportions of cure and sample sizes of each pair of groups. We found the following estimations for the study power: SR/AR=39.7%; SR/IL= 78.1%; AR/IL= 43.6%. The calculation was not added to the methodology section, but we added this in the Discussion section as a limitation (Page 35).

**Comment 1.2:** The population is defined except for the infecting species of *Leishmania*.

**Response 1.2:** Thanks for the recommendations. The characterization of the infectious species was added in methods and results (Pages 6 and 24).

**Comment 1.3:** Under exclusion criteria it mentions excluding for prior CL treatment, but does not give a time frame for this.

**Response 1.3:** Thanks for the comment. We added a phrase on Page 6.

**2.RESULTS**

**Comment 2.1:** There are over 100 data points missing on several variables (...)

**Response 2.1:** Thank you for your concern. Although we are a national reference centre for leishmaniasis treatment, data loss is a reality in retrospective studies. If we consider the amount of data information analyzed, the number of losses can be considered with little relevance (Page 35).

**Comment 2.2:** (...) the size of each arm is vastly different.

**Response 2.2:** Great observation. We emphasize the observational design of the study, which makes impossible to control the type of the performed treatment. In our reference centre, patients are mostly treated with the alternative regimen with meglumine antimoniate. When there was formal contraindication to systemic drugs, intralesional treatment has been traditionally indicated. Patients treated with the standard regimen, in general, were part of research projects. As explained previously, there was a relevant difference among the size of the three groups. This study limitation was discussed (Page 35).

**Comment 2.3:** Figure 1 and the text describing it do not match.

**Response 2.3:** Thanks for the comment. Figure 1 and its subtitle were corrected for better understanding. In addition, it was inserted at the beginning of the results session (Page 14). The exclusion criteria have been corrected (Page 9).

**3. CONCLUSIONS**

**Comment 3.1:** Given the size discrepancies between the arms it is hard to draw conclusions, however it clearly seems that the standard regimen of MA is poorly tolerated and the other regimens are likely good alternatives.

**Response 3.1:** Thanks for your coment. The main message of the study is that the standard regimen, despite having higher rates of effectiveness, has the disadvantage of higher toxicity. Alternative regimen and intralesional treatment were less effective, but better tolerated even in patients with comorbidities and formal contraindications to systemic meglumine antimoniate. Also, these regimens were effective in cases of no cure and recurrence of the disease. The percentage of evolution to mucosal leishmaniasis was reduced, with a follow-up time of at least three years.

**4. SUMMARY AND GENERAL COMMENTS**

**Comment 4.1:** This is a single-center, retrospective study or 592 patients with parasitologically diagnosed CL evaluating standard regimen MA, alternative regimen MA, and intralesional MA. The main findings are that all 3 arms have the same cure rate, but the standard regimen has more side effects and abandonment than the other arms. Given its retrospective nature the arms are not equal and the standard arm has only 46 subjects comparted to the alternative arm with 456, and the IL arm with 90. This makes it difficult to adequately compare the arms.

**Response 4.1:** We emphasize the observational design of the study, which makes impossible to control the type of the performed treatment. In our reference centre, patients are mostly treated with alternative regimen of meglumine antimoniate. In cases where there was a formal contraindication to systemic drug, intralesional treatment has been traditionally indicated. Patients treated with stantard regimen, in general, were part of research projects. As explained previously, there was a significant difference between the size of the three groups. This study limitation was discussed (Page 35).

**Comment 4.2:** Additionally variables are not available for many subjects with over 100 points missing on more than one variable used in the analysis.

**Response 4.2:** Thanks for the concern. We recognize the limitation of the study design. In retrospective analysis, data loss occur frequently. However, this is a robust cohort, with an expressive number of participants, amount of information and time of follow-up.

**Comment 4.3:** One major issue for the paper is that it discusses that the IL subjects return for more than one treatment, however the number of treatments and the average amount of MA used for each treatment is not discussed. It is fundamental to understand the amount of drug used to understand how AEs come into play in this type of study.

**Response 4.3:** Great suggestion. The requested calculations were described on the results session. Page 25.

**Comment 4.4:** Also, a major concern with giving local vs systemic treatment is the development of ML. They do mention that there is an initial evaluation for ML and those are excluded, but does not provide follow up to show if mucosal lesions developed.

**Response 4.4:** Great suggestion. The follow-up protocol at our reference center was more fully detailed (methods section, Page 9). In addition, the evolution to mucosal leishmaniasis was described in the results section (Page 19).

**Comment 4.5:** Also, the results say that IL has no treatment interruption, but how can this be defined when there is no pre-set schedule for giving the IL treatments?
**Response 4.5:** Thank you for the observation. Although there may be flexibility in the number of infiltrations performed, the interval between them was approximately fifteen days. The interruption of treatment connected to toxicity was associated with the extension of the interval between sessions due to an adverse event. Treatment completion due to therapeutic success occurred when epithelialization of the lesion(s) was verified.

**Comment 4.6:** Minor issues are Figure 1 not showing similar items to what is discussed in the text. It is unclear why there are 47 subjects excluded from the study, were they not treated in the study timeframe? Line 135 refers to 10 subjects being excluded which is not reflected in the figure.

**Response 4.6:** We are sorry for these mistakes. Figure 1 has been corrected. We removed from the figure the 6 cases of visceral leishmaniasis and included the 11 cases primarily treated with other therapeutic regimens than meglumine antimoniate, for better understanding. The 47 patients who were subtracted from the initial figure were not part of the study period (2000-2017). Line 135 referred to the exclusion of patients with the disseminated cutaneous form of the disease (≥ 10 lesions in more than one segment of the body).

**Comment 4.7:** Exclusion criteria do not match what is written in the text.

**Response 4.7:** Thanks for the concern. The exclusion criteria have been corrected (Page 9).

**Comment 4.8:** Also, using more commonly used English terms like 2 weeks instead of fortnight.

**Response 4.8:** Thanks for the suggestion. We corrected the grammar and spelling, improved the sentences, organized the text and also used a more precise language.

**----------------------------------------------------------------------------------------------------------**

**REVIEWER 2**

**5. METHODS**

**Comment 5.1:** There is no clear statement of the hypotheses of the study in the document.

**Response 5.1:** We apologize if the study hypothesis was not clear. We added this information on Page 6.

**Comment 5.2:** Because it is a retrospective study, there are marked differences in the size of the groups under evaluation, which potentially generates biases.

**Response 5.2:** Thanks for the comment. We added this limitation in Discussion (Page 35).

**6. RESULTS**

**Comment 6.1:** The analysis of the information is adequate but the results are only presented as percentages, without showing confidence intervals that allow the reader to better understand the findings.

**Response 6.1: Response 9.7:** We are sorry for this mistake. We added this information on results.

**Comment 6.2:** The authors should improve the clarity of the images in figure 6.

**Response 6.2:** Thanks for the concern. Figure 6 was rearranged for better understanding.

**7. CONCLUSIONS**

**Comment 7.1:** Yes partially. This is a retrospective observational study with limitations in its external validity, therefore its conclusions should be referential.

**Response 7.1:** Great observation. Despite being an observational study, the expressive number of patients analyzed allows the extrapolation of results to reference centres with similar conditions and introduces the discussion of treatments other than the standard dose recommended by the Brazilian guidelines, less toxic, reasonably effective and with a low rate of evolution to mucosal lesions after treatment (Page 34).

**Comment 7.2:** The authors highlight that the use of low-dose MA in the alternative (AR) and intralesional regimens is effective and has a significantly lower toxicity than the standard regimen (recommended in the Brazilian guidelines for the treatment of LC), but they do not mention that the regimen SR with MA was statistically better than the AR and IL regimens.

**Response 7.2:** We are sorry for the omission. It was corrected in abstract and discussion.

**Comment 7.3:** The authors should discuss the peculiar situation of the *Leishmania* strain that circulates in the State of RJ is sensitive to low doses of MA. There is information in the Brazilian literature that explains this type of response.

**Response 7.3:** We appreciated this recommendation. We included a paragraph on discussion. Page 34.

**Comment 7.4:** Authors should describe the limitations of the study.

**Response 7.4:** Thanks for the advice. Limitations are discussed.

**8. SUMMARY AND GENERAL COMMENTS**

**Comment 8.1:** Several publications in RJ have demonstrated the efficacy of IL therapy with MA in LC and of low doses of MA, although significantly lower CR than the SR regimen (Saheki et al 2017, ref # 16).

**Response 8.1:** Thanks for the comment. Although AR and IL treatment have been used predominantly in studies in the state of Rio de Janeiro, several authors demonstrate reasonable effectiveness of these schemes compared to the standard regimen, with the advantage of lower morbidity in the treatment of cutaneous leishmaniasis. We added this discussion on Page 34.

**Comment 8.2:** The greatest value of this study is to show as an alternative the AR regimen, traditionally used in INI / Fiocruz in RJ, with a cure rate of 84.3% versus 95.3% of SR, a lower effectiveness but with the advantage of being 10 times less toxic and have 3 times less abandons. This is a reasonable rate, especially for patients over 50 years of age, who have contraindications to the use of pentavalent antimonials due to the frequency and severity of adverse effects.

**Response 8.2:** We appreciate the pertinent observation. The main objective of this study was to demonstrate that the so-called alternative treatments (AR and IL) have reasonable effectiveness, with a lower percentage of toxicity.

**Comment 8.3:** This cohort has several peculiarities: (i) in INI / Fiocruz (ATL referral center) the treatment regimen of choice is AR (77% of the patients evaluated)...

**Response 8.3:** Great observation. In our reference centre, we follow standardized protocols for diagnosis and monitoring of patients that facilitate their follow-up for a long period. We used the alternative regimen as the first choice, after careful evaluation of the patient.

**Comment 8.4:** (ii) the SR (7.8%) and IL regimens (15.2%) are regularly used in LC patients with contraindications to the use of pentavalent antimonials by the systemic route (page 5, lines 113-177)....

**Response 8.4:** Thanks for the comment. In our centre, patients treated with the standard regimen, in general, were part of research projects and patients with contraindications to systemic MA were treated with intralesional infiltrations with reasonable effectiveness rate.

**Comment 8.5:** (iii) for the AR regimen they use low doses of MA (5mg Sb5+/Kg/day) for 30 consecutive days...

**Response 8.5:** Thank you for the observation. This is the alternative regime treatment protocol used by our centre and described national and internationally.

**Comment 8.6:** (iv) the majority of 552/592 patients (93.4%) were infected in the State of Rio de Janeiro

**Response 8.6:** Good point to comment. Although the majority of patients in this cohort are from the state of Rio de Janeiro, this centre receives patients from all states in the country. The discussion of this peculiarity has been added. Page 34.

**Comment 8.7:** (v) they have a surprising low abandonment rate at 3 (2.4%) and 6 (%) months, very different from the experience of other hospitals where the dropout rate is greater than 50% in this time (the researchers should make a brief description of the success of this retention).

**Response 8.7:** Thank you very much for the observation. We discussed the low dropout rates in discussion section. Page 29.

**9. LIMITATIONS**

**Comment 9.1:** The 3 treatment groups are not comparable, by nature how the treatment regimen was selected. Therefore, the reported effectiveness does not show superiority or inferiority of any of the 3 regimens used and the information is only referential.

**Response 9.1:** Great comment. This limitation was included. Page 35.

**Comment 9.2:** Due to the way in which patients have been managed (frequently MA has continued to be used between 2 to 4 times) without the authors having defined criteria for repeating or changing the regimen, it makes the effectiveness rates not comparable and difficult to interpret from the second scheme used.

**Response 9.2:** Thank you for the comment. We added a section on results “Outcomes in subsequent treatments” and additional information was compiled in data supporting S1 File, for better understanding. Page 24.

**Comment 9.3:** It is suggested not to use the term treatment failure, because in practice they have used the “no cure” criterion, which explains continuing to use the same drug (MA), although with a different regimen.

**Response 9.3:** Thanks for the comment. We changed the term therapeutic failure in the manuscript and in the figures.

**Comment 9.4:** Due to the nature of the study (retrospective observational), it has poor external validity that limits the extrapolation of conclusions for all of Brazil.

**Response 9.4:** Thanks for the concern. This limitation was discussed. Page 35.

**Comment 9.5:** The authors report that there are no statistical differences in the re-epithelialization rate (cure criterion at 3 months) between patients from Rio de Janeiro and other Brazilian states (lines 232-234), implicitly suggesting that the therapeutic response is similar. However, there is a clear contradiction with the results that the authors show in Figure 3, Picture G, where the healing rate (definitive cure criterion at 6 months) in is lower (p <0.001) in lesions acquired in other different states RJ.

**Response 9.5:** We apologize for the misunderstanding but these were different analyzes. We did not identified statistical differences between the crude rates (proportions) of epithelialization and complete healing regarding the origin of the patients (whether from the state of Rio de Janeiro or other Brazilian states). In the survival analysis, using Kaplan Meier method, patients from other states took longer to obtain complete healing. Page 32.

**Comment 9.6:** Furthermore, there are several reports in the literature with variations in the efficacy of Sb5 + in Leishmania (V.) braziliensis in different endemic areas of Brazil. Apparently the L. (V.) braziliensis strain circulating in RJ is susceptible to low doses of pentavalent antimonials. The authors do not comment on this evidence.

**Response 9.6:** Thanks for the meticulousness. It is known that the genetic variability of *Leishmania braziliensis* in the state of RJ is lower than in other regions of Brazil, but there are no concrete data that demonstrates greater susceptibility to treatment with MA in this particular state. Although trials with alternative dose of MA were performed only in RJ, the present study also did not show any difference in the efficacy of the performed treatments on patients from the state of RJ or those from other regions. The lack of standardization of concepts makes it difficult to compare results between studies carried out with different methodologies. The choice of analysis by protocol or by intention to treat as well as the interpretation of clinical cure criteria may explain, in part, the divergences in effectivity among different reports. Only with multicenter clinical trials we may answer this question definitively. This topic was added in discussion section (Page 34).

**Comment 9.7:** The cure rates (CR), only presented as percentages, should include the numerators and denominators, as well as the confidence intervals so that the reader can understand this complex study.

**Response 9.7:** We are sorry for this omission. We added this information in methods and the results sections.

**Comment 9.8:** Thus, the authors with the first AM scheme in the RA regimen report a CR of 84.3% and then mention that the cure rate for retreatment (understood as RA) was 69.2% (68 cases).

**Response 9.8:** Thank you for the comment. We added a section on results “Outcomes in subsequent treatments” and additional information was compiled in data supporting S1 File, for better understanding. Page 24.

**Comment 9.9:** Analyzing figure 3, only 56 patients received RA retreatment and a decrease in CR is observed with the 2nd treatment scheme 28/39 (71.8%), 3rd treatment 7/12 (53.8%) and 4th treatment 2/5 (40%). This message is important and not all treatment regimens should be grouped together because they have variable cure rates depending on the number of retreatments. It is also not advisable to mix IL regimens with RA because the CRs can vary. For this reason, the authors' description of retreatment (page 15) is confusing and does not have a clear message. Same comment for description of IL results.

**Response 9.9:** Thanks for the observation. We rewrote these results and the additional information was compiled in data supoorting (S1 File). Page 24.

**Comment 9.10:** The authors report epithelialization and healing time with no statistical difference between the 3 groups (lines 267-269), however, as they have different interventions (some more than one treatment scheme) the information is only referential. Based on these findings, the authors should recommend that prospective clinical studies be conducted.

**Response 9.10:** We thank you for your concern. In that section of the manuscript, the rates of epithelialization and healing among groups and the topography of lesions (cephalic segment and periarticular region) were compared. In the performed analyzes, there were no statistically significant differences among the groups. Even so, we recommend carrying out more controlled studies to evaluate alternative and IL treatments. Page 33.

**Comment 9.11:** Authors should have a study limitations section. This study has important limitations as it is a retrospective observational study.

**Response 9.11:** Great observation. We added the study limitations on Discussion.

**Comment 9.12:** The conclusion must be corrected, the effectiveness of the standard regimen with MA was statistically better than the AR and IL regimens, although with significantly less toxicity, an aspect that is not mentioned in the conclusion of the study abstract.

**Response 9.12:** Thanks for the observation. This information was added on Abstract.
